# Noncrop features and heterogeneity mediate overwintering bird diversity in agricultural landscapes of southwest China

**DOI:** 10.1002/ece3.6319

**Published:** 2020-04-29

**Authors:** Depin Li, Myung‐Bok Lee, Wen Xiao, Jia Tang, Zhengwang Zhang

**Affiliations:** ^1^ Key Laboratory for Biodiversity Science and Ecological Engineering College of Life Sciences Beijing Normal University Beijing China; ^2^ Institute of Eastern‐Himalaya Biodiversity Research Dali University Dali China; ^3^ Guangdong Key Laboratory of Animal Conservation and Resource Utilization Guangdong Public Laboratory of Wild Animal Conservation and Utilization Guangdong Institute of Applied Biological Resources Guangzhou China

**Keywords:** abundance, biodiversity conservation, crop heterogeneity, farmland birds, species richness

## Abstract

Farmland birds are of conservation concerns around the world. In China, conservation management has focused primarily on natural habitats, whereas little attention has been given to agricultural landscapes. Although agricultural land use is intensive in China, environmental heterogeneity can be highly variable in some regions due to variations in crop and noncrop elements within a landscape. We examined how noncrop heterogeneity, crop heterogeneity, and noncrop features (noncrop vegetation and water body such as open water) influenced species richness and abundance of all birds as well as three functional groups (woodland species, agricultural land species, and agricultural wetland species) in the paddy‐dominated landscapes of Erhai water basin situated in northwest Yunnan, China. Birds, crop, and noncrop vegetation surveys in twenty 1 km × 1 km landscape plots were conducted during the winter season (from 2014 to 2015). The results revealed that bird community compositions were best explained by amounts of noncrop vegetation and compositional heterogeneity of noncrop habitat (Shannon–Wiener index). Both variables also had a positive effect on richness and abundance of woodland species. Richness of agricultural wetland species increased with increasing areas of water bodies within the landscape plot. Richness of total species was also greater in the landscapes characterized by larger areas of water bodies, high proportion of noncrop vegetation, high compositional heterogeneity of noncrop habitat, or small field patches (high crop configurational heterogeneity). Crop compositional heterogeneity did not show significant effects neither on the whole community (all birds) nor on any of the three functional groups considered. These findings suggest that total bird diversity and some functional groups, especially woodland species, would benefit from increases in the proportion of noncrop features such as woody vegetation and water bodies as well as compositional heterogeneity of noncrop features within landscape.

## Graphical abstract

We found that bird community compositions were best explianed by compositional heterogeneity of noncrop habitat (Shannon‐Wiener index; Non‐cropH) and amount of noncrop vegetation (Non‐cropP).

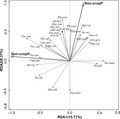

## 1 INTRODUCTION

Over the past 50 years, agricultural intensification has been one of the main drivers of biodiversity decline in both temperate and tropical regions (Amano et al., 2008; Donald, Green, & Heath, 2001; Haslem & Bennett, 2008; Ikin et al., 2014). Given the fact that the subtropical and tropical regions of developing countries have experienced the most intense transformations (Rudel et al., 2009), there is an urgent need to identify key factors that can minimize biodiversity loss in these areas.

Several studies have emphasized the importance of habitat or landscape heterogeneity (environmental heterogeneity, hereafter) for conserving or restoring biodiversity in agricultural landscapes (Batáry, Fischer, Báldi, Crist, & Tscharntke, 2011; Benton, Vickery, & Wilson, 2003; Fahrig et al., 2011). Environmental heterogeneity is measured primarily based on seminatural and natural elements such as compositional heterogeneity (diversity of these elements) and configurational heterogeneity (their spatial arrangement) at multiple spatial scales (Barbaro, Rossi, Vetillard, Nezan, & Jactel, 2007; Fahrig et al., 2011; Neumann, Griffiths, Foster, & Holloway, 2016). Increasing compositional heterogeneity, for example, increasing the richness and proportion of natural or seminatural habitats preserved in agricultural landscapes such as hedgerows, scrublands, riparian vegetation, woodlands, and ponds, can have a positive impact on biodiversity by providing different types of habitats or complementary resources for diverse plants and animals (Ricketts, 2001; Wethered & Lawes, 2003). A complex pattern of spatial arrangement (i.e., high configurational heterogeneity) of these seminatural and natural elements can increase the variability of microclimate conditions (Stein, Gerstner, & Kreft, 2014), facilitate animal movements between habitat patches (Fischer & Lindenmayer, 2002; Lawton et al., 2010), and improve resource accessibility of species (Fahrig et al., 2011). The amount of noncrop vegetation (trees, shrubs, and grassy/herbaceous vegetation) and other noncrop features (open water) as well as the characteristics of field margins also benefit biodiversity in agricultural landscapes (Amano et al., 2008; Gil‐Tena et al., 2015; Wilson et al., 2017). However, because increasing noncrop elements within farmlands would reduce the area of productive land, this may not be a desirable option in many cases (Fischer et al., 2008; Khoury et al., 2014). Crop or cropland heterogeneity has been proposed as an alternative strategy that may achieve both biodiversity conservation and agricultural productivity goals (Fahrig et al., 2011). Similar to the heterogeneity of noncrop elements (mostly seminatural/natural vegetation types), cropland heterogeneity can promote biodiversity when diverse crop types provide resources for different species (niche differentiation effects) or complementary resources (complementation effects) to meet the varying resource requirement of single species in space and time (Donald et al., 2001; Fahrig et al., 2011). Positive effects of crop compositional heterogeneity on the diversity of birds and insects are reported in several studies (Donald et al., 2001; Gottschalk et al., 2010; Palmu, Ekroos, Hanson, Smith, & Hedlund, 2014). Crop configurational heterogeneity may also positively affect biodiversity when there is a greater retention of seminatural habitats such as hedgerows, riparian corridors, and grassy strips at field edges or between fields (Evans, Burger, Riffell, & Smith, 2014; Weibull, Bengtsson, & Nohlgren, 2008). The positive relationship between crop configurational heterogeneity and biodiversity is often found (Collins & Fahrig, 2017; Fahrig et al., 2015). However, the effect of crop compositional heterogeneity shows inconsistent patterns, which may be associated with differences in farmland practices, variations in crop types, and the degree of agricultural intensity among study sites (Fahrig et al., 2015; Piha, Tiainen, Holopainen, & Vepsalanen, 2007; Tscharntke et al., 2016).

Agricultural land use in China has been continuously intensified since the 1970s due to high pressure on food security, leading to rapid loss of biodiversity (Baudry, Yu, & Cai, 1999; UNDP/GEF & MFPRC, 2005). However, biodiversity conservation being mainly focused on natural habitats. As little attention has given to agricultural landscape, there is a considerable lack of knowledge about the biodiversity–environment relationship in agricultural landscapes (Liu, Duan, & Yu, 2013).

Northwest Yunnan is located in the southern section of the Hengduan Mountains which are part of the south‐central China biodiversity hotspot (Myers, Mittermeier, Mittermeier, Fonseca, & Ket, 2000). This region is also situated on the western side of the flyway of migrant birds in China (Zhang & Yang, 1997). Similar to other agricultural areas of China, habitat degradation within traditional farmlands in northwest Yunnan has increased due to conversion to modern farming systems (e.g., construction of tractor roads) and industrial monocultures (e.g., massive garlic plantations in winter season), especially in plain areas, creating more homogeneous landscapes (Sun et al., 2017). Although this change could negatively affect biodiversity in the region, it is unknown how agricultural land uses and practices influence bird diversity.

We studied relationships between overwintering bird diversity and environmental characteristics in the agricultural landscapes of the Erhai basin situated in northwest Yunnan. In particular, our study aimed to examine how environmental heterogeneity (crop heterogeneity and noncrop heterogeneity) affects bird diversity in the plain areas of agricultural landscapes during the winter season. We considered birds as a target taxon because they play an important role in ecosystem functioning (Sekercioglu, 2006; Sodhi et al., 2005), are easy to sample, and can be a good environmental indicator (Sodhi et al., 2005). Given the positive biodiversity‐environmental heterogeneity relationship, we expected that both the total richness and abundance (including all birds; whole community) as well as the richness and abundance of three functional groups classified based on species' habitat preference would increase with increasing crop compositional heterogeneity, crop configurational heterogeneity (mean field patch size), and noncrop compositional heterogeneity. Furthermore, because noncrop vegetation can offer shelter, foraging, or nesting places for farmland birds (Benton et al., 2003; Fuller, Hinsley, & Swetnam, 2004), we also expected that the proportion of noncrop vegetation would have a positive effect on birds, particularly woodland and agricultural land species.

## 2 MATERIALS AND METHODS

### 2.1 Study area

**FIGURE 1:**
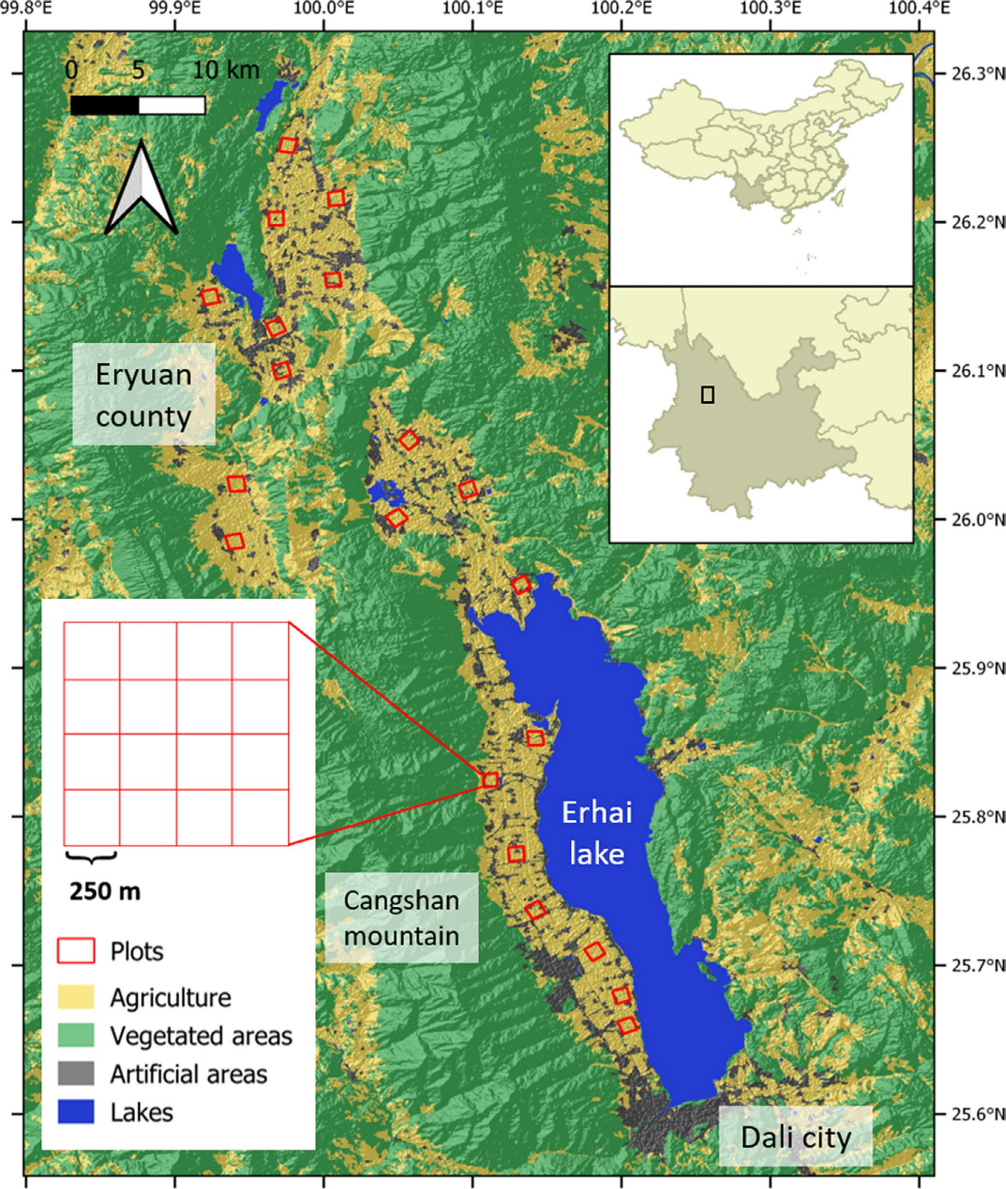
Location of the study region, Erhai water basin, northwest Yunnan, China. Each square represents a landscape plot, 1 km^2^ in size

This study was conducted in the plain areas of Erhai water basin (25°38′‐26°19′N, 99°55′‐100°13′E) situated in northwest Yunnan, China, spanning between Dali city in the south, a corridor lying between the Cangshan Mountain in the west and the Erhai Lake in the east, and part of Eryuan County in the north (Figure 1). The climate is subtropical plateau monsoon with annual precipitation of 1,000–1,200 mm (mainly in summer) and average annual temperature of 15.1°C (Ren, Yang, Wang, & Tang, 2011). The elevation of the study area varies between 1,970 and 2,200 m a. s. l. Although arable fields are predominant in the area, other land cover types such as grass vegetation, riparian forest, nursery garden (the area within farms that used by farmers to cultivate trees for commercial purpose), farm village, and urban area are common. Garlic (*Allium sativum*) is a main crop cultivated in the area, especially in the winter planting season (from October to April), representing over 40% of all crops. Other common crops are horse bean (*Vicia faba*), barley (*Hordeum* sp.), and romaine (*Lactuca* sp.). The native woody vegetation is largely composed of broadleaved deciduous forest and nursery garden vegetation, dominated by Chinese aspen (*Populus adenopoda*), Nepalese alder (*Alnus nepalensis*), willow (*Salix* sp.), and red rhododendron (*Rhododendron delavayi*). Vegetation cover at the grass layer is mainly occupied by annual meadow grass (*Poa annua*), love grass (*Eragrostis ferruginea*), stinging nettles (*Urtica laetevirens*), goosegrass (*Galium aparine*), and Bahama grass (*Cynodon dactylon*). Eighty‐seven bird species are known to be found in the agricultural landscapes near Dali City, fifty‐six of them belonging to the order Passeriformes (Han, Yan, & Deng, 2013). White Wagtail (*Motacilla alba*), Russet Sparrow (*Passer cinnamomeus*), Long‐tailed Shrike (*Lanius schach*), and Siberian Stonechat (*Saxicola maurus*) are the most dominant species (Han et al., 2013).

### 2.2 Data collection

#### 2.2.1 Survey design

We selected twenty 1 × 1 km landscape plots for bird surveys. The landscape plot size is large enough to include multiple landscape elements and small enough to allow replication and thorough sampling of all elements in each plot (Haslem & Bennett, 2008). The average distance between plot centers was 27.8 km (range 2.3–69.4 km). Landscape plots were selected by considering richness of land cover types and crop diversity (Table S1). In order to reduce the impacts of traffic and human activities, we avoided urban areas and main transportation lines.

#### 2.2.2 Bird surveys

To perform bird survey, we divided each plot into 16 blocks, 250 × 250 m in size each (Figure 1). Sample points were established at the center of each block, resulting in a total of 16 sample points per plot. Bird surveys were conducted four times (twice in the morning and twice in the evening for each landscape plot) between 25 October 2014 and 06 March 2015 by two observers, using a fixed 100‐m radius point count method (Bibby, Burgess, Hill, & Mustoe, 2000; Douglas et al., 2014). At each survey, the observer recorded all species seen or heard within a 100 m radius area surrounding a sample point, for 10 min. This duration is long enough to detect higher species numbers by avoiding double counts (Ralph, Droege, & Sauer, 1995). Birds flying over the sampling points were also recorded but excluded for analyses. Morning and evening surveys were conducted during the first 3 hr after sunrise and the last two and a half hours before sunset, respectively. We did not perform bird surveys under unsuitable weather conditions, such as rain, strong wind, or too high temperature. We alternated survey orders between two observers, between plots, and between points within a plot to ensure that landscape plots were sampled equally in morning and afternoon survey periods and to reduce a bias associated with observer (Haslem & Bennett, 2008). The two observers were well trained and had more than 4 years of experience in bird surveys, and thus, we assumed observer effect could be negligible in our study. Although a total of six points were located in forest habitat across three plots (1–3 points at three out of twenty plots) in this study, these forests had relatively low density of vegetation cover, especially understory vegetation. Thus, we also assumed that the habitat types at which sample points were located, for example, open habitat (farm field) versus forest habitat, would have a minimal effect on overall detectability of birds and our results. We followed recommendation from “A Checklist on the Classification and Distribution of the Birds of China (Third Edition)” for bird taxonomy and nomenclature (Zheng, 2017).

#### 2.2.3 Environmental variables

Within each landscape plot, we identified all crop and noncrop elements (eucalyptus, other woody vegetation, nonwoody vegetation, and water bodies) directly in the field and recorded them on a printed Google Earth image acquired between May 2014 and March 2015, but mostly between October and November 2014 (www.earth.google.com). The boundary between different crops within the same field was also determined using a Global Position System Receiver (Garmin 500) and marked on the printed image. All spatial information was digitized in Google Earth. Crop fields were delineated if there were visible field boundaries often covered with noncrop vegetation or if adjacent crop types were different regardless of the presence of noncrop vegetation on the boundary (Fahrig et al., 2015). Areas of each crop and noncrop feature were calculated using ArcGIS 10.1 (ESRI, 2011).

We found 23 different crops cultivated across twenty landscape plots. As a measure of crop diversity (crop compositional heterogeneity), we used Shannon–Wiener diversity index and calculated the index based on the genus of a crop (a total of 16 genera) because some crops were rare or their proportion was too small (Table S1; Lee & Goodale, 2018). The configurational heterogeneity of croplands was quantified by the mean patch size of all arable fields in a landscape plot (Fahrig et al., 2015; Josefsson, Berg, Hiron, Pärt, & Eggers, 2017). Considering the findings of previous studies (Heath, Candan, Karen, Rodd, & Sara, 2017; Lee & Goodale, 2018) and the characteristics of noncrop elements in our study area, we classified noncrop elements into five types associated with birds: eucalyptus vegetation (*Eucalyptus* spp.), other woody vegetation, nonwoody vegetation, water bodies (streams, lakes, and ponds), and old fallow (uncultivated land covered with weeds and crop stubble during study period). Composition heterogeneity of noncrop elements (noncrop habitat diversity; Shannon–Wiener diversity index) was calculated using the percent cover of each of the five types. We also summed the percent cover of eucalyptus, other woody vegetation, and nonwoody vegetation to indicate the amount of noncrop vegetation within a landscape plot. An overview of summary statistics for all variables used for analysis in final models is given in Table 1.

**TABLE 1:** Description of explanatory variables and response variables used for analysis

Variables	Description	Mean	*SD*	Min	Max
Explanatory variables					
Noncrop vegetation (Non‐cropP)	Sum of percent cover of eucalyptus, other woody vegetation (including trees planted in the nursery garden), and nonwoody vegetation	10.40	6.00	3.50	22.30
Water body	Total area (ha) of open water including stream, pond, and water reservoir for farming	0.42	0.69	0.00	2.89
Mean patch size (MPS, ha)	Mean field size	0.08	0.02	0.05	0.13
Crop Shannon's diversity (CropH)	Shannon–Wiener diversity index calculated based on the 16 genera of 23 different crops cultivated across our study area (Table S1 for the list of crops)	0.79	0.35	0.142	1.43
Noncrop habitat diversity (Non‐cropH)	Shannon–Wiener diversity index calculated using the proportional cover of five noncrop habitat types: eucalyptus, other woody vegetation (such as small forest patch dominated by Chinese aspen, alder, or willow), nonwoody vegetation, water body, and old fallow	1.10	0.24	0.50	1.53
Response variables					
Total species richness	Total number of species detected at least once during four visits	33.50	5.33	17.00	43.00
Abundance of total species	Mean number of all birds per visit	187.60	32.21	126.00	235.00
Woodland species richness	Total number of woodland species detected at least once during four visits	5.40	3.03	0.00	13.00
Abundance of woodland species	Mean number of birds of woodland species per visit	17.25	12.04	0.00	43.50
Agricultural land species richness	Total number of agricultural land species detected at least once during four visits	12.05	1.61	9.00	15.00
Abundance of agricultural land species	Mean number of birds of agricultural land species per visit	138.49	25.91	97.75	188.00
Agricultural wetland species richness	Total number of agricultural wetland species detected at least once during four visits	5.65	2.58	0.00	10.00
Abundance of agricultural wetland species	Mean number of birds of agricultural wetland species per visit	3.98	3.74	0.00	17.00

### 2.3 Data analysis

We categorized 86 species into five groups based on habitat preferences by referring to Zhao (2001), Amano et al. (2008), Katayama, Osawa, Amano, and Kusumotoa (2015), and “Handbook to the Birds of the World Alive” (http://www.hbw.com): agricultural land species (birds using dry farmland), agricultural wetland species (birds foraging in agricultural wetlands such as ponds and wet fields), woodland species (forest edge, open forest, and forest interior species), raptors (Falconiformes, Accipitriformes, and Strigiformes), and urban species (see Table S1 for habitat preferences of these 86 species). Raptor and urban species were included in whole community (total species richness and abundance) but not analyzed separately because of their low occurrence.

Each landscape plot (1 × 1 km in size) was considered as a sample unit for analysis, following the approach of previous studies (Amano et al., 2008; Douglas et al., 2014; Haslem & Bennett, 2008). For richness of total species and each ecological group, we included species detected at least once during surveys. Abundance was calculated by summing all counts within a plot and then dividing the sum by 4 visits, following the approach of other studies (Amano et al., 2008; Douglas et al., 2014). The distance (≥250 m) of sample points in each landscape plot was relatively close and survey used a 100 m radius, which may increase the risk of double counts (dependence between point counts within a plot) in our study. However, we considered that the effect of double counts was consistent across plots, because we used the same number of points in each landscape plot and all points were equally surveyed by each of two observers.

To examine how landscape characteristics affect species composition of birds in agricultural landscapes, we used ordination methods in Canoco (Windows Version 5.0; Smilauer & Leps, 2014). Detrended correspondence analysis (DCA) was run a priori in order to estimate the length of the composition gradient. The gradient length, that is, 1.690 for the first axis, indicated that the linear models of redundancy analysis (RDA) were appropriate (Ter Braak & Smilauer, 2002). We used a total of 86 species detected at least once during four visits and mean abundance of each species across four visits at each plot. Abundance data were Hellinger‐transformed to reduce double zero problems, as suggested by Legendre and Gallagher (2001) and Borcard, Gillet, and Legendre (2011). Forward stepwise model building approach in RDA in conjunction with Monte Carlo permutation test (1,000 random permutations) was used to identify environmental variables that better explain the variation of bird communities across landscape plots.

To examine the relationship between species diversity and landscape variables, generalized linear models (GLMs) were built with Poisson distribution for richness of woodland species and with Gaussian distribution for total species richness, agricultural land species richness, and abundance of each group. We used generalized least squares (GLS) model with VarExp for richness of agricultural wetland species, to deal with the violation of homoscedasticity. Following a multimodel inference approach (Burnham & Anderson, 2002), we constructed 24 models by a combination of all variables including null model (intercept‐only model) and ranked them based on the Akaike's information criterion corrected for small samples (AICc). A set of models, in which ΔAICc (difference in AICc between the best model and subsequent model) was <4, were considered to have equivalently strong empirical support and similar plausibility (Burnham & Anderson, 2002). We averaged the parameters of these selected models (ΔAICc < 4) and determined the relative importance of environmental variables using the sum of Akaike weights (**∑_Wi_**) of each model as described by Burnham and Anderson (2002). GLMs were performed using “glm” function in MASS package (Venables & Ripley, 2002) and model averaging using “model.avg” function in MuMIn package (Bartoń, 2017). GLS were conducted using “gls” function in “nlme” package (Pinheiro, 2019). These analyses were performed using R 3.4.1 (R development Core Team, 2017).

Before final RDA and GLM analyses, we calculated the nearest distance from an edge of each plot to the edge of large lake, large native forest patch, and the nearest urban area. We tested whether these distance variables could affect species richness and abundance of birds. None of the distance variables did show a significant effect on any response variable (Table S2). Thus, we concluded their effect could be negligible and did not include them in final models.

We also tested spatial dependency (whether bird observations from closer plots were more alike than plots further apart) using Moran's I test in the R “ape” package (Paradis et al., 2019) and found that spatial autocorrelation to be insignificant for all cases (*p* > .05; Table S4). Both correlation values and variance inflation factors (VIFs) were examined to check for collinearity. Pearson's correlation among explanatory variables was ≤|0.5|. VIFs were less than 4.0 for most of cases, except the relationship between richness of agricultural wetland species and the proportion of noncrop vegetation cover (VIF of Non‐cropP = 17.56). We excluded Non‐cropP from the model and recalculated the VIFs. VIFs of all variables in the model were below 4.0 except for water body (VIF = 5.23) (Table S4). Thus, we considered the multicollinearity of covariates in our data to be minimal.

## 3 RESULTS

### 3.1 Bird community

Of 86 bird species recorded, 36 species were migrants (Table S3). White Wagtail, Eurasian Tree Sparrow, oriental skylark (*Alauda gulgula*), and Siberian Stonechat were the most abundant species, comprising 60% of total abundance (sum of all birds found in 20 landscape plots).

**FIGURE 2:**
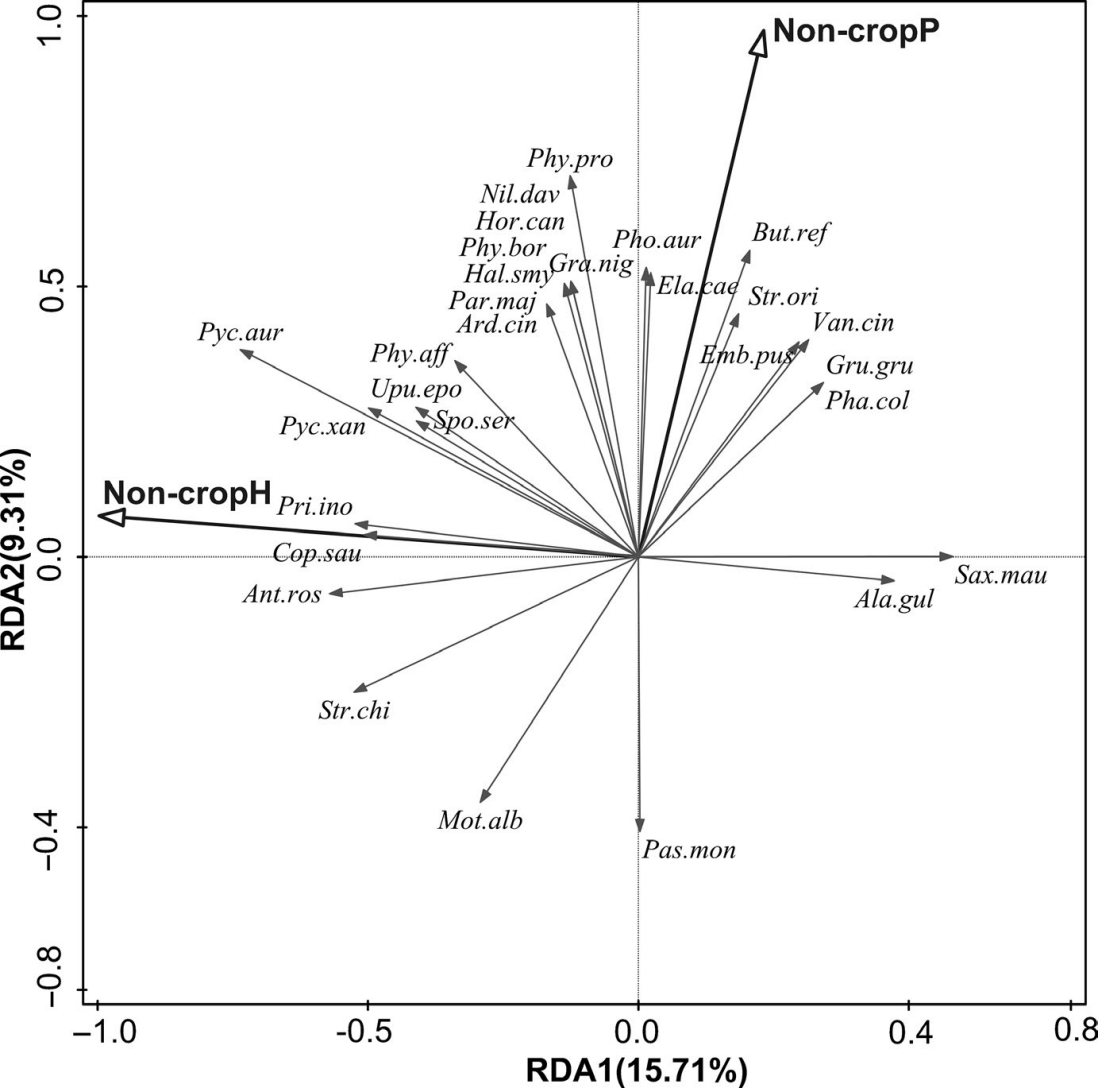
RDA biplot visualizing the associations between landscape variables and bird species composition. Biplot displays 30 species with the largest fit in the ordination space. Non‐cropH indicates noncrop habitat diversity. Non‐cropP is noncrop vegetation percentage per plot, which is sum of percent cover of eucalyptus, other woody vegetation (including nursery garden trees), and nonwoody vegetation. Species abbreviation consists of the first three letters in genus name and the first three letters in species scientific name (See Table S3 for full name). Only significant explanatory variables (*p* < .05) are included in ordination. Proportion of total variation explained by each axis is given in parenthesis

The effects of all landscape variables explained 34.4% (*F* = 1.47, *p* = .001) of the total variation in bird community composition based on the results from RDA. Forward stepwise selection identified two key explanatory variables explaining 25.02% of the total variation. The first axis (15.71% of total variation) and the second axis (9.31% of total variation) of RDA were associated with diversity of noncrop habitat type (Non‐cropH) and the proportion of noncrop vegetation cover (Non‐cropP) within a landscape plot, respectively (Figure 2). Non‐cropH was positively related to woodland species and some open species (species preferring open habitat, which include agricultural land species and agricultural wetland species), especially tickell's leaf warbler (*Phylloscopus affinis*), brown‐breasted bulbul (*Pycnonotus xanthorrhous*), sooty‐headed bulbul (*Pycnonotus aurigaster*), plain prinia (*Prinia inornata*), and oriental magpie robin (*Copsychus saularis*), and negatively to Siberian Stonechat and oriental skylark (Figure 2). Most woodland species, that is, Pallas's Leaf Warbler (*Phylloscopus proregulus*), Arctic Warbler (*Phylloscopus borealis*), Manchurian Bush Warbler (*Horornis canturians*), Fujian Niltava (*Niltava davidi*), Daurian Redstart (*Phoenicurus auroreus*), Cinereous Tit (*Parus cinereus*), and Oriental Turtle Dove (*Streptopelia orientalis*), and several open species such as Common Pheasant (*Phasianus colchicus*), grey‐headed lapwing (*Vanellus cinereus*), grey heron (*Ardea cinerea*), White‐throated Kingfisher (*Halcyon smyrnensis*), and Little Bunting (*Emberiza pusilla*) were correlated with high Non‐cropP, whereas White Wagtail and Eurasian Tree Sparrow were correlated with low Non‐cropP (Figure 2).

### 3.2 Bird diversity

**FIGURE 3:**
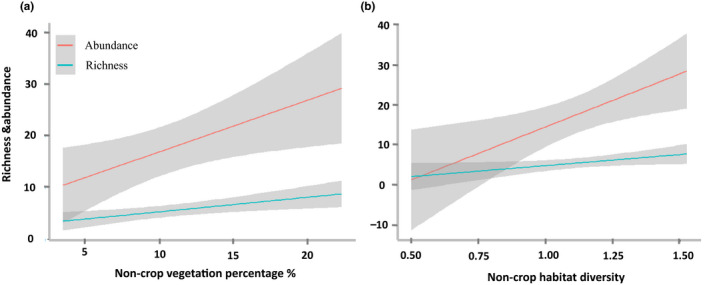
The effect of (a) proportion of noncrop vegetation (Non‐cropP) and (b) noncrop habitat diversity (Non‐cropH) on richness and abundance of woodland species. Grayed area represents a 95% confidence interval

**FIGURE 4:**
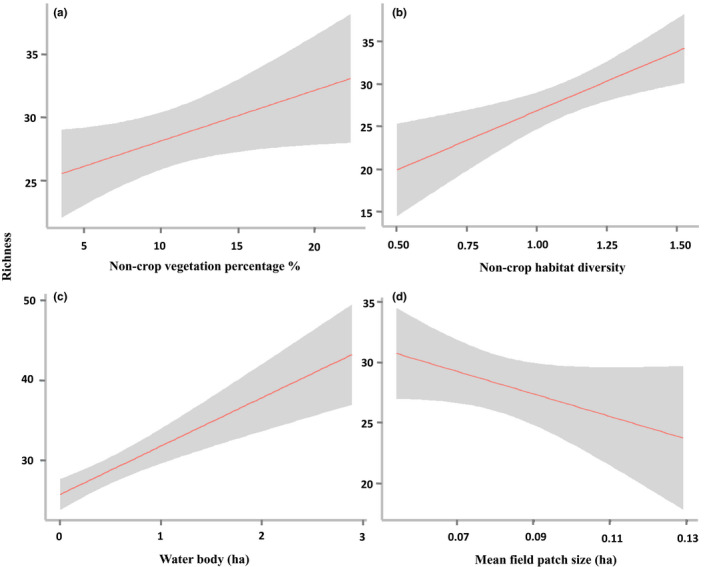
The effect of (a) proportion of noncrop vegetation (Non‐cropP), (b) noncrop habitat diversity (Non‐cropH), (c) area of water bodies, and (d) mean field patch size (MPS) on total species richness. Grayed area represents a 95% confidence interval

**FIGURE 5:**
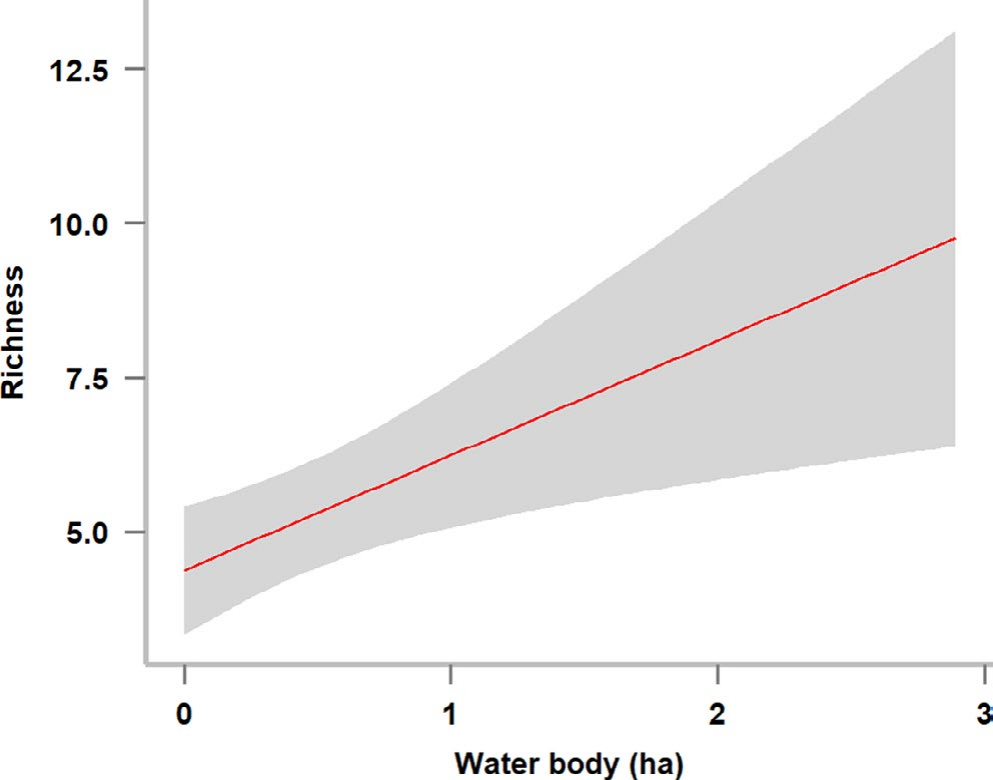
The effect of area of water bodies on the richness of wetland species. Grayed area represents a 95% confidence interval

Amount of noncrop vegetation (Non‐cropP) and noncrop habitat diversity (Non‐cropH) had positive impacts on richness and abundance of woodland species (Table 2 and Figure 3) as well as total species richness (Table 2 and Figure 4). Their 95% confidence intervals (CIs) did not contain 0, indicating a significant effect of Non‐CropP and Non‐cropH on those response variables. The total species richness and agricultural wetland species richness increased with increasing area of water bodies (Table 2, Figures 4 and 5). Agricultural land species did not show a strong association with landscape variables (Table 2).

**TABLE 2:** Model‐averaged parameter estimates, adjusted standard errors (*SE*), 95% confidence intervals (CI), and relative variable weight (*w_i_*)

Response variable	Parameter	Estimate	Adjusted *SE*	95% CI (Lower, Upper)	*_Wi_*
Species richness					
Total	Intercept	**28.30**	**0.66**	**27.01, 29.59**	
	Non‐cropP	**2.29**	**0.90**	**0.53, 4.05**	**0.90**
	Water body	**2.37**	**0.92**	**0.57, 4.17**	**1.00**
	Non‐cropH	**2.22**	**0.87**	**0.50, 3.95**	**1.00**
	CropH	NA	NA	NA	NA
	MPS	**−1.79**	**0.82**	**−3.39, −0.20**	**0.69**
Woodland species	Intercept	**1.53**	**0.12**	**1.30, 1.76**	
	Non‐cropP	**0.35**	**0.10**	**0.13, 0.56**	**1.00**
	Water body	NA	NA	NA	NA
	Non‐cropH	**0.39**	**0.14**	**0.11, 0.66**	**1.00**
	CropH	0.01	0.06	−0.19, 0.37	0.15
	MPS	−0.18	0.14	−0.46, 0.11	0.28
Agricultural land species	Intercept	**2.46**	**0.07**	**2.32, 2.60**	
	Non‐cropP	NA	NA	NA	NA
	Water body	0.04	0.07	−0.10, 0.17	0.15
	Non‐cropH	0.04	0.07	−0.10, 0.18	0.15
	CropH	0.01	0.07	−0.19, 0.37	0.13
	MPS	−0.03	0.07	−0.18, 0.11	0.14
Agricultural wetland species	Intercept	**5.69**	**0.51**	**4.69, 6.70**	
	Water body	**0.71**	**0.19**	**0.33, 1.10**	**0.95**
	Non‐cropH	0.67	0.56	−0.43, 1.76	0.23
	CropH	−0.56	0.30	−1.15, 0.03	0.26
	MPS	−0.68	0.39	−1.44, 0.09	0.37
Abundance					
Total	Intercept	**5.22**	**0.04**	**5.14, 5.31**	
	Non‐cropP	0.04	0.05	−0.05, 0.14	0.19
	Water body	0.01	0.05	−0.08, 0.10	0.08
	Non‐cropH	−0.01	0.05	−0.10, 0.08	0.08
	CropH	0.06	0.05	−0.03, 0.14	0.32
	MPS	−0.01	0.05	−0.10, 0.08	0.13
Woodland species	Intercept	**1.53**	**0.12**	**2.31, 2.86**	
	Non‐cropP	**0.35**	**0.10**	**0.12, 0.80**	**1.00**
	Water body	0.20	0.11	−0.53, 0.19	0.55
	Non‐cropH	**0.39**	**0.14**	**0.29, 0.99**	**1.00**
	CropH	NA	NA	NA	NA
	MPS	−0.18	0.14	−0.69, 0.02	0.28
Agricultural land species	Intercept	**4.92**	**0.04**	**4.84, 5.01**	
	Non‐cropP	0.05	0.05	−0.05, 0.13	0.17
	Water body	0.02	0.05	−0.08, 0.11	0.06
	Non‐cropH	−0.08	0.05	−0.17, 0.01	0.58
	CropH	0.06	0.04	−0.03, 0.15	0.25
	MPS	0.04	0.05	−0.07, 0.14	0.13
Agricultural wetland species	Intercept	**1.38**	**0.16**	**1.07, 1.70**	
	Non‐cropP	0.25	0.19	−0.12, 0.62	0.41
	Water body	0.23	0.17	−0.11, 0.57	0.33
	Non‐cropH	0.12	0.17	−0.21, 0.46	0.10
	CropH	−0.21	0.19	−0.57, 0.16	0.52
	MPS	−0.08	0.19	−0.45, 0.28	0.09

Note

Estimates of variables not included in results of model averaging based on a set of candidate models (ΔAICc < 4) were indicated as NA.

Abbreviations: CropH, crop diversity (crop compositional heterogeneity); MPS, mean field patch size (crop configurational heterogeneity); Non‐cropH, noncrop diversity; Non‐cropP, proportion (percentage) of noncrop elements; Water body, area of open water.

Mean field patch size (MPS) had negatively associated with total species richness (Table 2 and Figure 4), indicating greater species richness at small fields and the significant effect of crop configurational heterogeneity on avian diversity. However, crop diversity (CropH; crop compositional heterogeneity) did not affect species richness and abundance of whole community or functional groups, given its wide 95% CI across 0 (Table 2).

## 4 DISCUSSION

Two environmental heterogeneity variables (crop and noncrop heterogeneity) and different components of heterogeneity (compositional and configurational heterogeneity) were considered in our study. As we expected, we found strong effects of compositional heterogeneity of noncrop vegetation on total species richness and both richness and abundance of woodland species. The amount of noncrop vegetation also had an impact on these variables. However, the effects of crop heterogeneity varied depending on the component of heterogeneity: While crop configuration heterogeneity significantly affected the total species richness, crop composition heterogeneity did not show strong effects on richness and abundance of the whole community and the functional groups considered.

### 4.1 Factors shaping species communities

The compositional heterogeneity of noncrop habitats (Non‐cropH) was the most important landscape factor driving bird composition in the RDA (Figure 2). This result is consistent with the findings of previous studies that reported the positive associations of birds with noncrop habitat heterogeneity in agricultural landscapes (Atauri & de Lucio, 2001; Leyequién, de Boer, & Toledo, 2010). Amount of noncrop vegetation (Non‐cropP) also significantly influenced the composition of bird community: Woodland species and open habitat species (agricultural land species and agricultural wetland species) were positively related to Non‐cropP. These patterns support the importance of the noncrop vegetation to enhance avian diversity in agriculture landscapes (Berg, 2002; Gil‐Tena et al., 2015; Jakobsson & Lindborg, 2017).

Among a group of species, it is noteworthy that several agricultural wetland species, such as grey‐headed lapwing, grey heron, and White‐throated Kingfisher, were positively associated with Non‐cropP. While the positive effect of Non‐cropP on woodland species and agricultural land species is well documented (Haslem & Bennett, 2008; Isacch & Cardoni, 2011; Neumann et al., 2016), relatively few studies have examined the response of agricultural wetland species to Non‐cropP. In our study area, most patches of water body (open water) were often surrounded by nursery or orchard gardens or composed of some small pools within gardens and maintained wet ground because of frequent irrigation. The wet ground and small pools may host more plants and insects, which increase foraging opportunities for these agricultural wetland species.

### 4.2 Factors important for species richness and abundance

Our study shows that the amount of noncrop vegetation in agricultural landscapes can enhance bird diversity, especially richness and abundance of woodland species (Table 2; Figure 3). Noncrop vegetation in our study includes eucalyptus, non‐eucalyptus trees, shrubs, and grass vegetation at field margins or between fields and at roadsides. Noncrop vegetation, especially woody habitat, offers a wide range of benefits for birds by providing nesting and roosting sites and by increasing connectivity between patches (Benton et al., 2003; Fuller et al., 2004). Thus, the retention of natural or seminatural habitats is considered important for the conservation of birds (Evans et al., 2014; Lindsay et al., 2013) and arthropods (Duelli & Obrist, 2003; Billeter et al., 2008) in agricultural landscapes.

The areas of water bodies had a positive effect on richness of agricultural wetland species. Most farmland fields were drained because of dry farming (e.g., planting garlic) during the period of our survey. Orchard‐dominant matrix surrounding water bodies, small ponds, and wet ground can be used for foraging. In these situations, open water may play as a main habitat for agricultural wetland species. The positive effect of open water (e.g., the total area of ponds within a 1 × 1 km area) on wintering birds is also reported in paddies in Asia (Amano et al., 2008; Chan, Severinghaus, & Lee, 2007).

In general, the compositional heterogeneity of noncrop cover is expected to have a positive effect on biodiversity, because heterogeneous environment (e.g., diverse habitat types) can provide more niches or complementary resources for different species, facilitate resource use by maintaining supplemental habitats, and increase resource accessibility (Fahrig et al., 2011; Leibold et al., 2004). In our study, we found significantly positive effects of noncrop habitat diversity on total species richness and richness and abundance of woodland species, which are often observed in other avian studies (Gil‐Tena et al., 2015; Lee & Goodale, 2018; Redlich, Martin, Wende, & Steffan‐Dewenter, 2018).

With the same rationale, we also expected a positive relationship between crop heterogeneity and bird diversity. Our results showed that increasing crop configurational heterogeneity, that is, decreasing mean crop field size, could have a positive effect on total species richness (Table 2; Figure 4d), which partly supports our prediction. A similar pattern was also found in other studies (Fahrig et al., 2015; Josefsson et al., 2017). In our study, the influence of crop configurational heterogeneity may be associated with the characteristics of field boundaries or edges. Most of the boundaries between fields were covered with grassy vegetation and had thin linear woody features (shrub or tree). Such boundary conditions could benefit not only agricultural land species such as plain prinia, White Wagtail, and Rosy Pipit (*Anthus roseatus*) but also woodland species such as sooty‐headed bulbul, Red‐billed Starling (*Spodiopsar sericeus*), and brown‐breasted bulbul by providing foods or perching sites for these species (Josefsson et al., 2017; Vicker, Feber, & Fuller, 2009).

However, we did not find a clear pattern between crop diversity (crop compositional heterogeneity) and bird richness or abundance. Previous studies suggested that crop diversity may be an important part of environmental heterogeneity in simplified agricultural landscapes, especially where few noncrop vegetation patches remain (Josefsson et al., 2017; Tscharntke et al., 2016). In our landscape plot, the proportion of noncrop vegetation was relatively high (10.4 ± 6%) and field size was small (0.08 ± 0.02 ha). Birds may benefit more from noncrop vegetation than diverse crops.

### 4.3 Caveats

The findings of our study provide variable insights on environmental factors associated with diversity and composition of avian communities in agricultural landscapes. However, there are several caveats that may limit our understanding. First, we considered the Shannon–Wiener diversity index as a proxy of the crop compositional heterogeneity. This metric does not account for variations in crop composition and structure (e.g., crop height). Several recent studies reported a strong effect of crop composition and structural diversity on birds (Josefsson et al., 2017; Santana et al., 2017). Specific crop type can be more important for avian communities, particularly farmland birds than crop diversity per se (Redlich et al., 2018). The main crop in our landscape plots was garlic, which may not be favored by birds for foraging. Compared to garlic, barley could benefit birds more because it can provide food such as grains and insects for birds (Li, unpublished data). However, we do not know how different crops cultivated in our study area are functionally associated with birds. Second, the spatial scale of a landscape often determines the outcome of landscape–biodiversity studies (Gabriel et al., 2010; Jackson & Fahrig, 2015). We considered a plot size of 1 × 1 km to represent the landscape. Although this size is often used in bird studies (Amano et al., 2008; Fahrig et al., 2015; Haslem & Bennett, 2008), it is uncertain whether it is suitable to capture variations in environmental features and related responses of all birds in our study area. In addition, taxon like birds that are highly mobile may respond to crop diversity at larger spatial scales than ours, given that significant responses of birds have been reported at some studies performed at larger landscape scale than ours (Jackson & Fahrig, 2015). It is also uncertain whether landscape properties (e.g., crop and noncrop heterogeneity and features) would remain the same at larger or smaller scales than the 1 km^2^ scale. Third, the seasonal variations in characteristics of habitat and bird community composition may cause different responses of bird to environmental features in agricultural landscapes (Amano et al., 2008; Guyot, Arlettaz, Korner, & Jacot, 2017). Another our study indicates variations in community composition of birds and crop types between summer (breeding season) and winter (Li, unpublished data). Thus, we need future research that employs a multiple spatial and temporal scale approach and focuses on the functional relationships between bird species and crop composition.

## 5 CONCLUSIONS

Our findings highlight the importance of noncrop elements, especially the woody and grass vegetation features within landscape for conserving or maintaining total bird richness and both richness and abundance of woodland bird. The positive effects of the diversity of noncrop habitats and small field patch size (i.e., crop configurational heterogeneity) on total bird richness support the importance of environmental heterogeneity in agricultural landscapes for avian diversity conservation.

Field consolidation caused by the construction of standardized farmlands increasingly occurring in China as well as the land usage right circulation (a household having the right to contract for management of rural land transfers land management right to another household or economic organization) has reduced seminatural habitat and increased field size. This poses an important question for conservation management in agricultural landscapes: How does the field consolidation affect bird assemblages? Previous studies show that boundaries between fields are a key characteristic influencing birds (Evans et al., 2014), insects (Weibull et al., 2008), and plants (Benton et al., 2003). There is a need to quantify how birds and other taxa such as arthropods and plants respond to changes in remnant habitats and field size to develop appropriate conservation strategies in agricultural landscape in China. In addition, recent several studies indicated that practices and policies adopted in increasing nature habitat (tree or shrub) within or adjacent to croplands could enhance pest control service by providing habitat for birds (e.g., insectivores) but could also increase nesting or roosting habitat for granivores and frugivores that damage crop seeds and fruits (Gonthier et al., 2019; Pejchar et al., 2018). Therefore, it is important to consider both the services and disservices of birds when making management decisions.

## CONFLICT OF INTEREST

None declared.

## AUTHOR CONTRIBUTION

**Depin Li:** Conceptualization (supporting); Formal analysis (lead); Investigation (lead); Methodology (lead); Writing‐original draft (equal); Writing‐review & editing (lead). **Myung‐Bok Lee:** Conceptualization (supporting); Formal analysis (supporting); Writing‐original draft (equal); Writing‐review & editing (supporting). **Wen Xiao:** Conceptualization (supporting); Project administration (supporting). **Jia Tang:** Data curation (supporting); Project administration (supporting). **Zhengwang Zhang:** Conceptualization (lead); Project administration (lead); Writing‐original draft (supporting); Writing‐review & editing (supporting).

## Supporting information

Table S1‐S4

## Data Availability

All data from this study are available at Dryad, https://doi.org/10.5061/dryad.k3j9kd53g. Data obtained 6 documents.
