# Supplementary material for: Noncrop features and heterogeneity mediate overwintering bird diversity in agricultural landscapes of southwest China
Source: Ecol Evol. 2020 Apr 29;10(12):5815–28. doi: 10.1002/ece3.6319 (PMC7319240; doi:10.1002/ece3.6319)
Supplement: Supplementary file 1 — Table S1‐S4 [file ECE3-10-5815-s001.doc]

**Non-crop features and heterogeneity mediate bird diversity in agricultural landscapes of Northwest Yunnan, China**

Depin Li, Myung-Bok Lee, WenXiao, Jia Tang, Zhengwang Zhang ^＊^

Table S1. List of crops planted at agricultural lands in the Erhai water basin, Yunnan, China, during winter planting season. Values represent a relative percent cover, i.e., total area of each crop (sum of each crop (non-crop element) area per sites)/total area of each landscape plot × 100 %. C: crop species, N: non-crop elements.

| Plot number | Crop/non-crop types | Genus | Percentage | Note |
| --- | --- | --- | --- | --- |
| 1 | Barley | Hordeum | 0.085 | C |
| 1 | Carrot | Daucus | 0.253 | C |
| 1 | Celery | Apium | 0.049 | C |
| 1 | Chinese cabbage | Brassica | 5.634 | C |
| 1 | Chive | Allium | 0.049 | C |
| 1 | Cos lettuce | Lactuca | 56.29 | C |
| 1 | Garlic | Allium | 9.368 | C |
| 1 | Green onion | Brassica | 0.070 | C |
| 1 | Green turnip | Raphanus | 1.931 | C |
| 1 | Leek | Allium | 0.035 | C |
| 1 | Peppermint | Mentha | 0.131 | C |
| 2 | Broccoli | Brassica | 0.242 | C |
| 2 | Carrot | Daucus | 1.296 | C |
| 2 | Celery | Apium | 0.270 | C |
| 2 | Chinese cabbage | Brassica | 3.613 | C |
| 2 | Cos lettuce | Lactuca | 45.72 | C |
| 2 | Garlic | Allium | 6.930 | C |
| 2 | Green onion | Brassica | 1.470 | C |
| 2 | Green turnip | Raphanus | 14.87 | C |
| 2 | Green vegetable | Brassica | 0.084 | C |
| 3 | Barley | Hordeum | 0.486 | C |
| 3 | Broccoli | Brassica | 0.112 | C |
| 3 | Carrot | Daucus | 0.671 | C |
| 3 | Celery | Apium | 0.085 | C |
| 3 | *Chenopodium* sp. | Chenopodium | 1.078 | C |
| 3 | Chinese cabbage | Brassica | 0.172 | C |
| 3 | Coriander | Coriandrum | 0.044 | C |
| 3 | Cos lettuce | Lactuca | 0.463 | C |
| 3 | Garlic | Allium | 7.915 | C |
| 3 | Green onion | Brassica | 0.308 | C |
| 3 | Green turnip | Raphanus | 0.153 | C |
| 3 | Green vegetable | Brassica | 0.134 | C |
| 3 | Horse bean | Vicia | 53.06 | C |
| 3 | *Lavandula* sp. | Lavandula | 0.147 | C |
| 3 | Leek | Allium | 0.205 | C |
| 3 | Lettuce | Lactuca | 0.068 | C |
| 3 | Oilseed rape | Brassica | 0.673 | C |
| 3 | Peas | Pisum | 0.191 | C |
| 3 | Peppermint | Mentha | 5.113 | C |
| 4 | Barley | Hordeum | 2.121 | C |
| 4 | Chinese cabbage | Brassica | 0.120 | C |
| 4 | Cos lettuce | Lactuca | 0.125 | C |
| 4 | Garlic | Allium | 37.66 | C |
| 4 | Horse bean | Vicia | 34.32 | C |
| 4 | *Lavandula* sp. | Lavandula | 0.206 | C |
| 4 | Oilseed rape | Brassica | 1.130 | C |
| 5 | Barley | Hordeum | 0.565 | C |
| 5 | Chinese cabbage | Brassica | 0.050 | C |
| 5 | Garlic | Allium | 30.05 | C |
| 5 | Horse bean | Vicia | 23.38 | C |
| 5 | Oilseed rape | Brassica | 0.418 | C |
| 6 | Chinese cabbage | Brassica | 0.055 | C |
| 6 | Garlic | Allium | 36.34 | C |
| 6 | Horse bean | Vicia | 43.77 | C |
| 7 | Barley | Hordeum | 0.251 | C |
| 7 | Garlic | Allium | 73.34 | C |
| 7 | Green turnip | Raphanus | 0.060 | C |
| 7 | Green vegetable | Brassica | 0.047 | C |
| 7 | Horse bean | Vicia | 1.532 | C |
| 7 | Oilseed rape | Brassica | 0.270 | C |
| 7 | Peas | Pisum | 0.066 | C |
| 7 | Ryegrass | Lolium | 0.063 | C |
| 8 | Garlic | Allium | 65.66 | C |
| 8 | Horse bean | Vicia | 7.923 | C |
| 9 | Barley | Hordeum | 0.060 | C |
| 9 | Garlic | Allium | 84.35 | C |
| 9 | Green onion | Brassica | 0.037 | C |
| 9 | Green turnip | Raphanus | 0.087 | C |
| 9 | Horse bean | Vicia | 1.657 | C |
| 9 | *Lavandula* sp. | Lavandula | 0.121 | C |
| 9 | Oilseed rape | Brassica | 0.036 | C |
| 9 | Ryegrass | Lolium | 0.239 | C |
| 10 | Barley | Hordeum | 0.176 | C |
| 10 | Garlic | Allium | 59.20 | C |
| 10 | Horse bean | Vicia | 18.58 | C |
| 10 | *Medicago* sp. | Medicago | 0.208 | C |
| 10 | Ryegrass | Lolium | 0.700 | C |
| 11 | Barley | Hordeum | 0.696 | C |
| 11 | Garlic | Allium | 14.02 | C |
| 11 | Horse bean | Vicia | 48.64 | C |
| 11 | *Medicago* sp. | Medicago | 6.284 | C |
| 11 | Oilseed rape | Brassica | 0.152 | C |
| 11 | Ryegrass | Lolium | 1.367 | C |
| 12 | Barley | Hordeum | 12.72 | C |
| 12 | Garlic | Allium | 15.38 | C |
| 12 | Horse bean | Vicia | 21.66 | C |
| 12 | *Medicago* sp. | Medicago | 11.01 | C |
| 12 | Oilseed rape | Brassica | 0.021 | C |
| 12 | Ryegrass | Lolium | 1.263 | C |
| 13 | Barley | Hordeum | 0.818 | C |
| 13 | Carrot | Daucus | 0.056 | C |
| 13 | Chinese cabbage | Brassica | 0.029 | C |
| 13 | Garlic | Allium | 58.05 | C |
| 13 | Horse bean | Vicia | 9.942 | C |
| 13 | *Medicago* sp. | Medicago | 2.183 | C |
| 13 | Oilseed rape | Brassica | 0.053 | C |
| 13 | Ryegrass | Lolium | 0.664 | C |
| 14 | Barley | Hordeum | 8.485 | C |
| 14 | Garlic | Allium | 43.64 | C |
| 14 | Horse bean | Vicia | 9.460 | C |
| 14 | *Medicago* sp. | Medicago | 8.474 | C |
| 15 | Garlic | Allium | 6.766 | C |
| 15 | Green onion | Brassica | 2.814 | C |
| 15 | Green vegetable | Brassica | 0.083 | C |
| 15 | Horse bean | Vicia | 72.00 | C |
| 15 | *Medicago* sp. | Medicago | 0.069 | C |
| 16 | Barley | Hordeum | 0.543 | C |
| 16 | Blueberry | Vaccinium | 12.12 | C |
| 16 | Broccoli | Brassica | 0.159 | C |
| 16 | Chinese cabbage | Brassica | 0.955 | C |
| 16 | Garlic | Allium | 1.338 | C |
| 16 | Green onion | Brassica | 0.027 | C |
| 16 | Green turnip | Raphanus | 0.076 | C |
| 16 | Horse bean | Vicia | 27.54 | C |
| 16 | Oilseed rape | Brassica | 2.619 | C |
| 16 | Peas | Pisum | 0.840 | C |
| 17 | Barley | Hordeum | 6.697 | C |
| 17 | Garlic | Allium | 6.645 | C |
| 17 | Green vegetable | Brassica | 0.190 | C |
| 17 | Horse bean | Vicia | 66.01 | C |
| 17 | *Medicago* sp. | Medicago | 0.561 | C |
| 18 | Barley | Hordeum | 0.226 | C |
| 18 | Garlic | Allium | 0.190 | C |
| 18 | Horse bean | Vicia | 2.559 | C |
| 18 | *Medicago* sp. | Medicago | 15.61 | C |
| 18 | Oilseed rape | Brassica | 1.143 | C |
| 19 | Barley | Hordeum | 4.619 | C |
| 19 | Blueberry | Vaccinium | 10.10 | C |
| 19 | Carrot | Daucus | 0.605 | C |
| 19 | Garlic | Allium | 0.166 | C |
| 19 | Horse bean | Vicia | 25.63 | C |
| 19 | *Medicago* sp. | Medicago | 0.098 | C |
| 19 | Oilseed rape | Brassica | 14.91 | C |
| 19 | Ryegrass | Lolium | 0.882 | C |
| 20 | Barley | Hordeum | 7.684 | C |
| 20 | Garlic | Allium | 52.78 | C |
| 20 | Horse bean | Vicia | 13.71 | C |
| 20 | *Medicago* sp. | Medicago | 7.715 | C |
| 20 | Ryegrass | Lolium | 0.582 | C |
| 1 | Euclyptus | - | 0.275 | N |
| 1 | Grass vegetation | - | 6.124 | N |
| 1 | old fallow | - | 3.967 | N |
| 1 | Other woody vegetation | - | 4.098 | N |
| 1 | Water body | - | 1.257 | N |
| 2 | Euclyptus | - | 1.026 | N |
| 2 | Grass vegetation | - | 3.378 | N |
| 2 | old fallow | - | 1.077 | N |
| 2 | Other woody vegetation | - | 1.668 | N |
| 2 | Water body | - | 0.192 | N |
| 3 | Euclyptus | - | 0.324 | N |
| 3 | Grass vegetation | - | 4.815 | N |
| 3 | old fallow | - | 2.816 | N |
| 3 | Other woody vegetation | - | 14.74 | N |
| 3 | Water body | - | 0.109 | N |
| 4 | Euclyptus | - | 0.490 | N |
| 4 | Grass vegetation | - | 4.000 | N |
| 4 | old fallow | - | 3.607 | N |
| 4 | Other woody vegetation | - | 5.171 | N |
| 5 | Grass vegetation | - | 7.105 | N |
| 5 | old fallow | - | 0.371 | N |
| 5 | Other woody vegetation | - | 1.527 | N |
| 5 | Water body | - | 0.719 | N |
| 6 | Euclyptus | - | 1.692 | N |
| 6 | Grass vegetation | - | 2.379 | N |
| 6 | old fallow | - | 0.403 | N |
| 6 | Other woody vegetation | - | 2.156 | N |
| 7 | Euclyptus | - | 0.251 | N |
| 7 | Grass vegetation | - | 5.212 | N |
| 7 | old fallow | - | 6.252 | N |
| 7 | Other woody vegetation | - | 1.206 | N |
| 8 | Euclyptus | - | 0.645 | N |
| 8 | Grass vegetation | - | 1.833 | N |
| 8 | old fallow | - | 1.621 | N |
| 8 | Other woody vegetation | - | 1.866 | N |
| 8 | Water body | - | 0.776 | N |
| 9 | Euclyptus | - | 0.528 | N |
| 9 | Grass vegetation | - | 3.822 | N |
| 9 | old fallow | - | 1.424 | N |
| 9 | Other woody vegetation | - | 1.660 | N |
| 10 | Euclyptus | - | 0.912 | N |
| 10 | Grass vegetation | - | 1.388 | N |
| 10 | old fallow | - | 1.143 | N |
| 10 | Other woody vegetation | - | 2.484 | N |
| 10 | Water body | - | 0.178 | N |
| 11 | Euclyptus | - | 0.136 | N |
| 11 | Grass vegetation | - | 3.069 | N |
| 11 | old fallow | - | 4.185 | N |
| 11 | Other woody vegetation | - | 7.412 | N |
| 11 | Water body | - | 0.241 | N |
| 12 | Euclyptus | - | 0.022 | N |
| 12 | Grass vegetation | - | 8.543 | N |
| 12 | old fallow | - | 8.630 | N |
| 12 | Other woody vegetation | - | 12.97 | N |
| 12 | Water body | - | 0.958 | N |
| 13 | Grass vegetation | - | 3.262 | N |
| 13 | old fallow | - | 6.600 | N |
| 13 | Other woody vegetation | - | 7.213 | N |
| 13 | Water body | - | 0.553 | N |
| 14 | Euclyptus | - | 0.301 | N |
| 14 | Grass vegetation | - | 6.727 | N |
| 14 | old fallow | - | 12.92 | N |
| 14 | Other woody vegetation | - | 5.603 | N |
| 15 | Grass vegetation | - | 2.270 | N |
| 15 | old fallow | - | 6.143 | N |
| 15 | Other woody vegetation | - | 2.335 | N |
| 15 | Water body | - | 0.388 | N |
| 16 | Euclyptus | - | 0.310 | N |
| 16 | Grass vegetation | - | 6.922 | N |
| 16 | old fallow | - | 13.50 | N |
| 16 | Other woody vegetation | - | 15.04 | N |
| 16 | Water body | - | 2.772 | N |
| 17 | Grass vegetation | - | 9.413 | N |
| 17 | old fallow | - | 6.519 | N |
| 17 | Other woody vegetation | - | 1.163 | N |
| 18 | Euclyptus | - | 0.436 | N |
| 18 | Grass vegetation | - | 15.98 | N |
| 18 | old fallow | - | 49.77 | N |
| 18 | Other woody vegetation | - | 4.009 | N |
| 19 | Grass vegetation | - | 7.753 | N |
| 19 | old fallow | - | 33.16 | N |
| 19 | Other woody vegetation | - | 0.080 | N |
| 20 | Grass vegetation | - | 0.676 | N |
| 20 | old fallow | - | 5.340 | N |
| 20 | Other woody vegetation | - | 2.828 | N |
| 20 | Water body | - | 0.087 | N |

Table S2. The results of simple regression distance variables with richness and abundance of each bird group, and redundancy analysis (RDA) with bird community composition and all variables. DSU, distance of edge of each plot to edge of the nearest urban; DSW, distance of edge of each plot to edge of the nearest large water body; DSF, distance of edge of each plot to edge of the nearest native forest patch; Non-crop, proportion (percentage) of non crop vegetation; Non-CropH, non-crop habitat diversity (Shannon-Wiener index).

| Bird group/bird community  composition | |  | Estimate | T value | SE | P value |
| --- | --- | --- | --- | --- | --- | --- |
| woodland species | richness | Intercept | 4.12 | 1.62 | 2.55 | 0.022 |
|  |  | DSU | 0.90 | 0.56 | 1.60 | 0.129 |
|  |  | DSW | 0.06 | 0.26 | 0.22 | 0.827 |
|  |  | DSF | -0.37 | 0.64 | -0.57 | 0.577 |
|  | Abundance | Intercept | 2.42 | 0.51 | 4.74 | 0.000 |
|  |  | DSU | 0.24 | 0.18 | 1.36 | 0.192 |
|  |  | DSW | -0.05 | 0.08 | -0.57 | 0.579 |
|  |  | DSF | -0.06 | 0.20 | -0.31 | 0.758 |
| Total species | richness | Intercept | 27.28 | 2.65 | 10.29 | 0.000 |
|  |  | DSU | 1.92 | 0.92 | 2.08 | 0.054 |
|  |  | DSW | -0.60 | 0.43 | -1.40 | 0.182 |
|  |  | DSF | -0.28 | 1.05 | -0.27 | 0.791 |
|  | abundance | Intercept | 163.57 | 16.76 | 9.76 | 0.000 |
|  |  | DSU | 7.05 | 5.83 | 1.21 | 0.245 |
|  |  | DSW | 3.00 | 2.71 | 1.11 | 0.285 |
|  |  | DSF | 3.87 | 6.66 | 0.58 | 0.569 |
| Agricultural land  species | Richness | Intercept | 11.49 | 0.74 | 15.53 | 0.000 |
|  |  | DSW | -0.25 | 0.12 | -2.05 | 0.057 |
|  |  | DSU | 0.46 | 0.26 | 1.80 | 0.090 |
|  |  | DSF | 0.09 | 0.29 | 0.32 | 0.752 |
|  | Abundance | Intercept | 136.31 | 14.15 | 9.63 | 0.000 |
|  |  | DSU | 0.36 | 4.93 | 0.07 | 0.943 |
|  |  | DSW | 0.96 | 2.29 | 0.42 | 0.681 |
|  |  | DSF | 2.44 | 5.62 | 0.43 | 0.670 |
| Agricultural  wetland species | Richness | Intercept | 4.93 | 1.20 | 4.11 | 0.001 |
|  |  | DSW | -0.29 | 0.19 | -1.49 | 0.155 |
|  |  | DSU | 0.42 | 0.42 | 1.00 | 0.332 |
|  |  | DSF | 0.23 | 0.48 | 0.48 | 0.636 |
|  | Abundance | Intercept | 1.29 | 0.37 | 3.46 | 0.003 |
|  |  | DSU | 0.05 | 0.13 | 0.35 | 0.733 |
|  |  | DSW | -0.05 | 0.06 | -0.82 | 0.424 |
|  |  | DSF | 0.11 | 0.15 | 0.75 | 0.463 |
|  |  |  | Explains% | |  | P value |
| Bird community  composition |  | DSW | 8.40 |  |  | 0.058 |
|  |  | DSF | 6.00 |  |  | 0.140 |
|  |  | DSU | 4.50 |  |  | 0.302 |

Table S3. Lists of bird species used for analysis. Species are classified according to habitat preferences. Agricultural land species (birds using dry farmland), agricultural wetland species (birds foraging agricultural wetland, such as ponds and wet fields), woodland species (e.g., forest edge, open forest and forest interior species, raptor (Falconiformes, Accipitriformes and Strigiformes) and urban species. V: migrating bird species; R: resident bird species.

| **Common name^1^** | **Scientific name** | **Species abbreviation** | **Habitat preference^2^** | **Distribution** |
| --- | --- | --- | --- | --- |
| Eurasian Crag Martin※ | *Ptyonoprogne rupestris* | *Pty.rup* | Agricultural land | V |
| Crested Myna | *Acridotheres cristatellus* | *Acri.cri* | Agricultural land | R |
| White Wagtail | *Motacilla alba* | *Mot.alb* | Agricultural land | R |
| Scaly-breasted Munia | *Lonchura punctulata* | *Lonc.pun* | Agricultural land | R |
| Plain Prinia | *Prinia inornata* | *Pri.ino* | Agricultural land | R |
| Eurasian Hoopoe | *Upupa epops* | *Upu.epo* | Agricultural land | R |
| Rosy Pipit | *Anthus roseatus* | *Ant.ros* | Agricultural land | V |
| Siberian Stonechat | *Saxicola maurus* | *Sax.mau* | Agricultural land | R |
| Black-headed Greenfinch | *Chloris ambigua* | *Chl.amb* | Agricultural land | R |
| Brown Shrike | *Lanius cristatus* | *Lan.cri* | Agricultural land | V |
| Grey-backed Shrike | *Lanius tephronotus* | *Lan.tep* | Agricultural land | R |
| Burmese Shrike | *Lanius collurioides* | *Lan.col* | Agricultural land | R |
| Long-tailed Shrike | *Lanius schach* | *Lan.sch* | Agricultural land | R |
| Common Crane | *Grus grus* | *Grus.gru* | Agricultural land | V |
| White-cheeked Starling | *Spodiopsar cineraceus* | *Stu.cin* | Agricultural land | V |
| Black-faced Bunting | *Emberiza spodocephala* | *Embe.spo* | Agricultural land | V |
| Russet Sparrow | *Passer cinnamomeus* | *Pas.cin* | Agricultural land | R |
| Richard’s Pipit | *Anthus richardi* | *Ant.ric* | Agricultural land | V |
| Little Bunting | *Emberiza pusilla* | *Emb. Pus* | Agricultural land | V |
| Oriental Skylark | *Alauda gulgula* | *Ala.gul* | Agricultural land | R |
| Ring-necked Pheasant | *Phasianus colchicus* | *Pha.col* | Agricultural land | R |
| Bluethroat | *Luscinia svecica* | *Lus.sve* | Agricultural land | V |
| Pied Bushchat | *Saxicola caprata* | *Sax.cap* | Agricultural land | R |
| Crested Bunting | *Melophus lathami* | *Mel.lat* | Agricultural land | R |
| Little Egret | *Egretta garzetta* | *Egr.gar* | Agricultural wetland | R |
| White-breasted Waterhen | *Amaurornis phoenicurus* | *Ama. Pho* | Agricultural wetland | R |
| Green Sandpiper | *Tringa ochropus* | *Tri.och* | Agricultural wetland | V |
| Grey Heron | *Ardea cinerea* | *Arde.cin* | Agricultural wetland | R |
| Chinese Pond Heron | *Ardeola bacchus* | *Arde.bac* | Agricultural wetland | R |
| Northern Lapwing | *Vanellus vanellus* | *Vane.van* | Agricultural wetland | V |
| Grey-headed Lapwing | *Vanellus cinereus* | *Van.cin* | Agricultural wetland | V |
| Yellow Wagtail | *Motacilla flava* | *Mot. Fla* | Agricultural wetland | V |
| Citrine Wagtail | *Motacilla citreola* | *Mot.cit* | Agricultural wetland | V |
| Gray Wagtail | *Motacilla cinerea* | *Mot.cin* | Agricultural wetland | V |
| Pacific Golden Plover | *Pluvialis fulva* | *Plu. Ful* | Agricultural wetland | V |
| Little Ringed Plover | *Charadrius dubius* | *Cha.dub* | Agricultural wetland | V |
| Wood Sandpiper | *Tringa glareola* | *Tri.gla* | Agricultural wetland | V |
| Cattle Egret | *Bubulcus ibis* | *Bub.ibi* | Agricultural wetland | R |
| Common Snipe | *Gallinago gallinago* | *Gal.gal* | Agricultural wetland | V |
| Plumbeous Water Redstart | *Rhyacornis fuliginosa* | *Rhy. Ful* | Agricultural wetland | R |
| White-throated Kingfisher | *Halcyon smyrnensis* | *Hal.smy* | Agricultural wetland | R |
| Common Moorhen | *Gallinula chloropus* | *Gal.chl* | Agricultural wetland | R |
| Green-winged Teal | *Anas crecca* | *Ana.cre* | Agricultural wetland | V |
| Mallard | *Anas platyrhynchos* | *Ana.pla* | Agricultural wetland | V |
| Common Kingfisher | *Alcedo atthis* | *Alc.att* | Agricultural wetland | R |
| Black-crowned Night Heron | *Nycticorax nycticorax* | *Nyc.nyc* | Agricultural wetland | R |
| Manchurian Bush Warbler | *Horornis canturians* | *Cet.can* | Woodland | V |
| Sooty-headed Bulbul | *Pycnonotus aurigaster* | *Pyc.aur* | Woodland | R |
| White-browed Laughingthrush | *Garrulax sannio* | *Gar.san* | Woodland | R |
| Spot-breasted Scimitar Babbler | *Erythrogenys gravivox* | *Ery. gra.* | Woodland | R |
| Daurian Redstart | *Phoenicurus auroreus* | *Pho.aur* | Woodland | V |
| Great Tit | *Parus major/cinereus* | *Par.maj* | Woodland | R |
| Black Drongo | *Dicrurus macrocercus* | *Dic.mac* | Woodland | V |
| Black-collared Starling | *Gracupica nigricollis* | *Gra.nig* | Woodland | R |
| Himalayan Bluetail | *Tarsiger rufilatus* | *Tar.ruf* | Woodland | R |
| Yellow-browed Warbler | *Phylloscopus inornatus* | *Phyl.ino* | Woodland | V |
| Brown-breasted Bulbul | *Pycnonotus xanthorrhous* | *Pyc.xan* | Woodland | R |
| Oriental White-eye | *Zosterops palpebrosus* | *Zos.pal* | Woodland | R |
| Ashy Drongo | *Dicrurus leucophaeus* | *Dic.leu* | Woodland | V |
| Chestnut-tailed Starling | *Sturnia malabarica* | *Stu.mal* | Woodland | R |
| Blue-fronted Redstart | *Phoenicuropsis frontalis* | *Pho. Fro* | Woodland | R |
| Blue Rock Thrush | *Monticola solitarius* | *Mon.sol* | Woodland | R |
| Oriental Turtle Dove | *Streptopelia orientalis* | *Stre.ori* | Woodland | R |
| Olive-backed Pipit | *Anthus hodgsoni* | *Ant.hod* | Woodland | V |
| Red-billed Starling | *Spodiopsar sericeus* | *Spo.ser* | Woodland | R |
| Eurasian Wryneck | *Jynx torquilla* | *Jyn.tor* | Woodland | V |
| Yellow-streaked Warbler | *Phylloscopus armandii* | *Phy.arm* | Woodland | V |
| White-throated Fantail | *Rhipidura albicollis* | *Rhi.alb* | Woodland | R |
| Black-browed Tit | *Aegithalos bonvaloti* | *Aeg.bon* | Woodland | R |
| Tickell’s Leaf Warbler | *Phylloscopus affinis* | *Phyl.aff* | Woodland | V |
| Darjelling Woodpecker | *Dendrocopos darjellensis* | *Den.dar* | Woodland | R |
| Pallas’s Leaf Warbler | *Phylloscopus proregulus* | *Phy.pro* | Woodland | V |
| Arctic Warbler | *Phylloscopus borealis* | *Phy.bor* | Woodland | V |
| Fujian Niltava | *Niltava davidi* | *Nil.dav* | Woodland | R |
| Rufous-vented Yuhina | *Yuhina occipitalis* | *Yuh.occ* | Woodland | R |
| Hen Harrier | *Circus cyaneus* | *Cir.cya* | Raptors | V |
| Short-eared Owl | *Asio flammeus* | *Asi.fla* | Raptors | V |
| Black-winged Kite | *Elanus caeruleus* | *Ela.cae* | Raptors | R |
| Common Kestrel | *Falco tinnunculus* | *Fal.tin* | Raptors | R |
| Common Buzzard | *Buteo refectus* | *But.ref* | Raptors | V |
| Pied Harrier | *Circus melanoleucos* | *Circ.mel* | Raptors | V |
| Common Magpie | *Pica pica* | *Pic.pic* | Urban | R |
| Barn Swallow | *Hirundo rustica* | *Hir.rus* | Urban | R |
| Eurasian Tree Sparrow | *Passer montanus* | *Pas.mon* | Urban | R |
| House Sparrow | *Passer domesticus* | *Pas.dom* | Urban | V |
| Spotted Dove | *Streptopelia chinensis* | *Stre.chi* | Urban | R |
| Oriental Magpie Robin | *Copsychus saularis* | *Cop.sau* | Urban | R |

1. Species are listed in taxonomic order following Guan-mei Zheng (2017)
2. Bird habitat categorizations based on Zhao (2001), Amano *et al*. (2008), Katayama *et al*.(2015) and Handbook to the Bird of Word Alive (http://ww.hbw.com)

※ Species was not included in analysis because of being recorded once time and flying over plot.

Table S4. Summary of average models relating spatial variation in richness and abundance of bird species to landscape variables. In each case we provide 95% confidence intervals, Moran’s I test p value and variance inflation factors (VIF). Variables excluding zero within 95% are in bold. Abbreviations: Non-crop, proportion (percentage) of non crop vegetation; CropH, crop diversity (Shannon-Wiener index); Non-CropH, non-crop habitat diversity (Shannon-Wiener index); MPS, mean field size.

| **Response** | **Parameter** | **Moran's I test p value** | **VIF** |
| --- | --- | --- | --- |
| Total Species richness (R^2^=0.84) | Intercept | 0.26 |  |
|  | Non-cropP |  | 1.90 |
|  | Water body |  | 1.69 |
|  | Non-CropH |  | 1.71 |
|  | CropH |  | 1.35 |
|  | MPS |  | 1.63 |
| Total Species abundance (R^2^=0.16) | Intercept | 0.62 |  |
|  | Non-cropP |  | 1.90 |
|  | Water body |  | 1.69 |
|  | Non-CropH |  | 1.71 |
|  | CropH |  | 1.35 |
|  | MPS |  | 1.63 |
| woodland species richness (R^2^=0.63) | Intercept | 0.76 |  |
|  | Non-cropP |  | 3.09 |
|  | Water body |  | 2.29 |
|  | Non-CropH |  | 1.84 |
|  | CropH |  | 1.71 |
|  | MPS |  | 1.59 |
| Woodland species abundance (R^2^=0.73) | Intercept | 0.71 |  |
|  | Non-cropP |  | 1.90 |
|  | Water body |  | 1.69 |
|  | Non-CropH |  | 1.71 |
|  | CropH |  | 1.35 |
|  | MPS |  | 1.63 |
| Agricultural land species richness (R^2^=0.15) | Intercept | 0.32 |  |
|  | Non-cropP |  | 1.99 |
|  | Water body |  | 1.75 |
|  | Non-CropH |  | 1.72 |
|  | CropH |  | 1.37 |
|  | MPS |  | 1.63 |
| Agricultural land species abundance (R^2^=0.26) | Intercept | 0.70 |  |
|  | Non-cropP |  | 1.90 |
|  | Water body |  | 1.69 |
|  | Non-CropH |  | 1.71 |
|  | CropH |  | 1.35 |
|  | MPS |  | 1.63 |
| Agricultural wetland species richness | Intercept | 0.42 |  |
|  | Non-cropP |  | § |
|  | Water body |  | 5.23 |
|  | Non-CropH |  | 1.93 |
|  | CropH |  | 1.92 |
|  | MPS |  | 3.43 |
| Agricultural wetland species abundance (R^2^=0.28) | Intercept | 0.44 |  |
|  | Non-cropP |  | 1.90 |
|  | Water body |  | 1.69 |
|  | Non-CropH |  | 1.71 |
|  | CropH |  | 1.35 |
|  | MPS |  | 1.63 |

**Reference**

Amano, T., Kusumoto Y., Tokuoka, Y., Yamada, S., Kim, E., & Yamamoto S. (2008). Spatial and temporal variations in the use of rice-paddy dominated landscapes by birds in Japan. Biological Conservation, 141:1704-1716. https://doi.org/10.1016/j.biocon.2008.04.012

Katayama, N., Osawa, T., Amano, T., & Kusumotoa, Y. (2015). Are both agricultural intensification and farmland abandonment threats to biodiversity ? A test with bird communities in paddy-dominated landscapes. Agriculture, Ecosystems and Environment, 214, 21-30. https://doi.org/10.1016/j.agee.2015.08.014.

Zhao, Z.J. (2001). A Handbook of the Birds of China. [M]. Jilin: Jilin Science and Technology Press. (in Chinese).

Zheng, G.M. (2017). A Checklist on the Classification and Distribution of the Birds of China (Second Edition) [M]. Beijing: China Science Press. (in Chinese).
